# Growth rate mediates hidden developmental plasticity of female yellow dung fly reproductive morphology in response to environmental stressors

**DOI:** 10.1111/ede.12396

**Published:** 2022-01-24

**Authors:** Richard J. Walters, David Berger, Wolf U. Blanckenhorn, Luc F. Bussière, Patrick T. Rohner, Ralf Jochmann, Karin Thüler, Martin A. Schäfer

**Affiliations:** ^1^ Department of Evolutionary Biology and Environmental Studies University of Zurich Zurich Switzerland; ^2^ Centre for Environmental and Climate Research Lund University Lund Sweden; ^3^ Evolutionary Biology Centre University of Uppsala Uppsala Sweden; ^4^ Biological and Environmental Sciences University of Stirling Stirling Scotland UK; ^5^ Biology and Environmental Sciences University of Gothenburg Gothenburg Sweden; ^6^ Department of Biology Indiana University Bloomington Indiana USA

**Keywords:** artificial selection, body size, developmental stability, Diptera, fluctuating asymmetry, growth rate, morphology, mortality, phenotypic plasticity, post‐copulatory sexual selection, spermatheca, survival, temperature

## Abstract

Understanding how environmental variation influences even cryptic traits is important to clarify the roles of selection and developmental constraints in past evolutionary divergence and to predict future adaptation under environmental change. Female yellow dung flies (*Scathophaga stercoraria*) typically have three sperm storage compartments (3S), but occasionally four (4S). More spermathecae are thought to be a female adaptation facilitating sperm sorting after mating, but the phenotype is very rare in nature. We manipulated the flies' developmental environment by food restriction, pesticides, and hot temperatures to investigate the nature and extent of developmental plasticity of this trait, and whether spermatheca expression correlates with measures of performance and developmental stability, as would be expected if 4S expression is a developmental aberration. The spermathecal polymorphism of yellow dung fly females is heritable, but also highly developmentally plastic, varying strongly with rearing conditions. 4S expression is tightly linked to growth rate, and weakly positively correlated with fluctuating asymmetry of wings and legs, suggesting that the production of a fourth spermatheca could be a nonadaptive developmental aberration. However, spermathecal plasticity is opposite in the closely related and ecologically similar *Scathophaga suilla*, demonstrating that overexpression of spermathecae under developmental stress is not universal. At the same time, we found overall mortality costs as well as benefits of 4S pheno‐ and genotypes (also affecting male siblings), suggesting that a life history trade‐off may potentially moderate 4S expression. We conclude that the release of cryptic genetic variation in spermatheca number in the face of strong environmental variation may expose hidden traits (here reproductive morphology) to natural selection (here under climate warming or food augmentation). Once exposed, hidden traits can potentially undergo rapid genetic assimilation, even in cases when trait changes are first triggered by random errors that destabilize developmental processes.

## Highlights

- Female yellow dung flies naturally vary in number of sperm storage compartments (3S or 4S).
- This spermathecal polymorphism is strongly heritable but also developmentally plastic.
- 4S expression is linked to growth rate and weakly correlated with fluctuating asymmetry, so potentially a developmental aberration.
- There are mortality costs as well as benefits for 4S phenotypes, suggesting adaptive life‐history trade‐offs.
- Spermathecal plasticity differs in the closely related and ecologically similar *Scathophaga suilla*.
- Environmental changes can expose hidden traits with initially no function to natural selection.

## Graphical abstract

Female yellow dung flies (*Scathophaga stercoraria*) express 3 or 4 sperm storage organs, the related *S. suilla 2* or 3 (right). Is this a developmental aberration, or an adaptive trait for sperm sorting? Environmental change (heat, pesticides, abundant food) augments developmental errors and induces spermathecal variation in proportion to growth rate, which can expose previously hidden traits to natural selection to produce a new beneficial function.

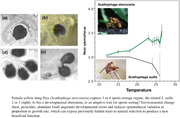

## 1 INTRODUCTION

Trait evolution occurs through the interplay between neutral and selective processes to ultimately produce morphological diversity by modifying underlying developmental mechanisms (West‐Eberhard, 2003). Traditionally, trait divergence was viewed to be mainly caused by (disruptive) natural selection followed by reinforcement upon secondary contact (Dobzhansky, 1937; Mayr, 1963), although recently the role of sexual selection has received more attention, especially for traits related to reproduction (Arnqvist & Rowe, 2005; Birkhead & Møller, 1998; Eberhard, 1996; Panhuis et al., 2001; Simmons, 2001). However, traits also evolve neutrally. Sex‐specific traits tend to accumulate more deleterious mutations under selection‐mutation balance because they are exposed to purging selection only half as often as the traits expressed in both sexes (De Visser et al., 2003; Mank & Ellegren, 2009; Van Dyken & Wade, 2010). Moreover, genetic correlations mediated by functional constraints and/or genetic linkage regularly shape evolutionary trajectories (Dobzhansky & Holz, 1943; Eberhard, 1996; Mayr, 1963; Moczek, 2009). Reproductive organs may thus be less evolutionarily stable and more susceptible to diversification via genetic drift (Civetta & Singh, 1998; De Visser et al., 2003; Van Dyken & Wade, 2010). Developmental plasticity can play a pivotal role for adaptation and divergence in novel environments, but can also constrain adaptive evolution by biasing trait expression towards certain developmental trajectories in favor of others (Chevin et al., 2010; Lande, 2009; Pfennig et al., 2010; Uller et al., 2018). Understanding precisely how environmental variation influences even cryptic internal morphological traits is therefore important both to clarify the roles of selection and developmental constraints in past evolutionary divergence and to predict future adaptation under environmental change.

**Figure 1:**
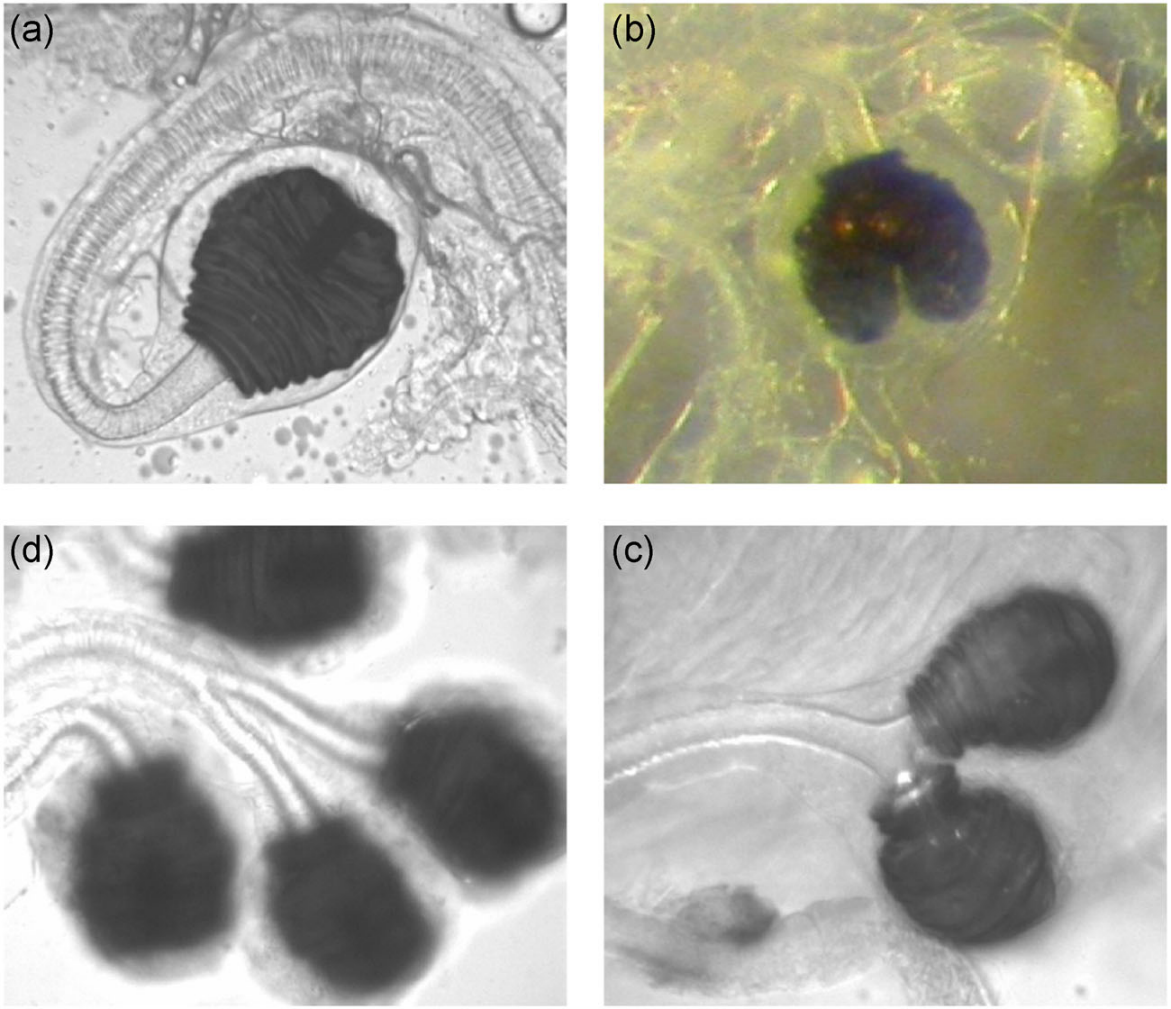
Various stages in the expression of 4S phenotypes: (a) normal singlet spermatheca (b) slightly invaginated, (c) fully split doublet with one duct, and (d) 4S phenotype with separate ducts [Color figure can be viewed at wileyonlinelibrary.com]

Intraspecific polymorphisms are particularly well suited for studying the evolutionary and developmental processes underlying evolutionary change. Reproductive traits of insects are very well studied in this regard (e.g., Arnqvist & Rowe, 2005; Parker, 1970; Pitnick et al., 1999; Simmons, 2001). As a prominent example, female sperm storage compartments have long been known to vary within and across various insect species (e.g., Dybas & Dybas, 1981; Jones & Ludlam, 1965; Kamimura, 2004; Minder et al., 2005; Pitnick et al., 1999; Presgraves et al., 1999; Puniamoorthy et al., 2010; Singh et al., 2006; Sturtevant, 1926). Spermathecae are sclerotized compartments of many female arthropods for storing sperm over extended periods of time, which tend to covary with other female but also male reproductive structures (Dybas & Dybas, 1981; Minder et al., 2005; Pitnick et al., 1999; Thüler et al., 2011). As one of the best‐studied species in the context of sexual selection (Simmons et al., 2020), female yellow dung flies *Scathophaga stercoraria* (Diptera: Scathophagidae) typically possess three (3S; one singlet and one doublet), but sometimes four spermathecae (4S; two doublets; Berger et al., 2011; Demont et al., 2021; Schäfer et al., 2013; Simmons et al., 1999; Ward et al., 2008; Ward, 2000). Intermediate phenotypes with bifurcated spermathecae and shared spermathecal ducts document the developmental transition between these alternative reproductive phenotypes (Ward et al., 2008; Figure 1). Sperm competition experiments provided evidence that multiple sperm storage organs can be advantageous during post‐copulatory sexual selection, and differential sperm storage and use has been repeatedly demonstrated in *S. stercoraria* and other insects using phenotypic or molecular markers (Bussière et al., 2010; Demont et al., 2012, 2021; Firman et al., 2017; Hellriegel & Bernasconi, 2000; Hellriegel & Ward, 1998; Lüpold et al., 2016; Manier et al., 2013; Otronen et al., 1997; Simmons et al., 2020; Snow & Andrade, 2005; Ward, 1998, 2000, 2007). As an additional spermatheca tends to reduce female fecundity, a trade‐off between any genetic benefits through female choice and life‐history costs has been suggested to maintain the spermathecal dimorphism within natural populations (Ward, 2007; Ward et al., 2008). However, the relative importance of neutral versus selective mechanisms for the evolution of this structure still remains unclear, as the 4S phenotype is rarely expressed in nature and therefore remains largely hidden from selection (Berger et al., 2011; cf. McGuigan & Sgrò, 2009), and growth rate strongly influences spermathecal expression (Schäfer et al., 2013). Genetic variation underlying the 4S phenotype can thus be cryptic, and alternatively this variation may, at least initially, merely represent stochastic developmental errors linked to rapid immature growth and decanalized development (Berger et al., 2011; Hadorn & Garber, 1944; Schäfer et al., 2013; Singh et al., 2006; Wexelson, 1928), which later upon exposure to selection may or may not become an adaptation in particular environments.

Here, we comprehensively investigate the plasticity of 4S expression in yellow dung flies exposed to a range of stressful environments during development to elucidate whether the production of a fourth spermatheca could be adaptive in the above sense or not. We first present hitherto unpublished results of laboratory spermathecal selection lines to document the extent of heritable versus plastic effects in spermathecal expression (Thüler, 2009; cf. Berger et al., 2011). By manipulating the flies' developmental environment in a variety of ways (by food (=dung) restriction, parasiticides, or hot temperatures) to impede or facilitate larval resource acquisition, development, and growth, we broadly assessed the nature and extent of variation in 4S expression (cf. McGuigan & Sgrò, 2009). We additionally monitored further life history and fitness traits (Dmitriew, 2011; Roff, 1997), notably pupal and total egg‐to‐adult survival, plus a common indicator of developmental instability, fluctuating asymmetry (FA) of paired morphological traits (Floate & Coughlin, 2010; Hosken et al., 2000; Palmer, 2000; Palmer & Strobeck, 1986; Parsons, 1992; Polak, 2003; Polak & Tomkins, 2013; Van Valen, 1962; Vøllestad et al., 1999; Whitlock, 1996). This specifically addresses the hypothesis that 4S (over)expression in yellow dung flies may represent a genetic aberration (as suggested for *Drosophila melanogaster*: Hadorn & Garber, 1944; Mather & Harrison, 1949; Singh et al., 2006; Wexelson, 1928), that is, a stochastic, nonadaptive consequence of systemic developmental instability, rather than an (ancestral) adaptation mediated by life‐history trade‐offs. We further present comparative data for the closely related and ecologically similar *S. suilla* (see Bernasconi et al., 2001) to demonstrate that spermathecal overexpression is no necessary consequence of accelerated growth, and that this type of (mostly hidden) developmental plasticity has evolved differently at least in two (but likely more) related taxa (cf. Minder et al., 2005).

## 2 MATERIALS AND METHODS

### 2.1 Artificial selection

From 65 yellow dung fly pairs originally collected at our experimental farm in Fehraltorf, Switzerland (N47°23′, E8°44′; the parental generation 1), selection lines were established by assigning emerged female flies to one of two regimes depending on whether they had three or four spermathecae, as determined retrospectively by dissection of the mother after having produced a clutch of eggs (see below). We randomly subdivided the subsequent (laboratory) generation 2 in each spermathecal category into three replicate lines (for a total of six lines). All adult flies were maintained individually in the laboratory under standard conditions in 100 ml glass bottles with ad libitum sugar, water, and *Drosophila* spp. as prey, at population densities of ca. 25–50 females (i.e., families) per generation per line. To produce the next generation, we randomly paired males and females within replicate lines by avoiding sibling matings, and raised a random subset of ca. 10–15 offspring (on average half of them being females) from the first clutch of each mother. We retroactively (see above) only forwarded offspring with the appropriate number of spermathecae (3S or 4S) to set up the next generation, spread as equitably as possible across all mothers of the previous generation. Offspring groups were always reared in full‐sibling groups in plastic containers with overabundant dung (see Section 2.3 and Blanckenhorn et al. [2010] for more detailed standard rearing and holding methods). At the end of artificial selection, males and females from the eighth generation were dissected to take pictures of their genital tracts with a microscope‐mounted camera (cf. Figure 1). Following an earlier, similar selection regime (Ward, 2000), we in parallel held unselected flies from the same population in our laboratory, whose spermathecae were however not monitored regularly, as we already knew that the 4S phenotype remains roughly stable across generations under laboratory conditions (see also Berger et al., 2011). There is hence no formal control treatment to report.

### 2.2 Dissections

Previously frozen females were dissected by carefully removing the posterior portion of the female reproductive tract from the body of the female by grasping the genital valves with forceps and tearing them from the abdomen. We could so separate the various internal reproductive female structures (bursa copulatrix, common oviduct, spermathecae and their ducts, accessory glands and their ducts, etc.) under a binocular microscope (Leica MZ‐12, Leica Microsystems GmbH) to photograph them (cf. Thüler et al., 2011). Using these photographs, we measured various internal structures of interest (spermatheca area/volume, spermatheca duct length, accessory gland area, and accessory gland duct length) as well as female hind‐tibia length as an index of body size, using ImageJ (Schneider et al., 2012; http://rsb.info.nih.gov/ij/).

### 2.3 Experimental treatments to manipulate fly growth, development, morphology, and survival

We exploited data from three separate *S. stercoraria* laboratory studies conducted at various temperatures to maximize their range of temperature exposure. In all cases, the adult parental flies originally stemmed from the same farm in Fehraltorf (cf. above) that were subsequently bred for 2–4 generations in the laboratory using standard methods (see Blanckenhorn et al., 2010, 2018, for details). In general, full‐sib family groups of 5–15 (mainly 10) offspring of laboratory‐mated females were reared in replicate plastic containers with (typically) overabundant (>2 g per larva; Amano, 1983) homogenized and previously frozen cow dung, thus minimizing larval competition. While two of the original experimental designs (splitting broods among mainly temperature environments) served to assess quantitative genetic variation, we here mainly considered phenotypic effects. One previously reported common garden rearing that also included *S. suilla* was performed at (always constant) 12, 18, and 24°C (Bauerfeind et al., 2018; Blanckenhorn et al., 2018; Schäfer et al., 2018), and additionally 26°C. A further, previously unpublished phenotypic laboratory study raised flies in half‐degree intervals at high temperatures from 24 to 26.5°C to approach the upper lethal limit of *S. stercoraria* (with no flies surviving beyond this temperature; cf. Blanckenhorn et al., 2014; Ward & Simmons, 1990). A more encompassing third study included a total of six treatments, to maximize the resulting range of growth rates: two temperatures (15 and 23°C) crossed with two food treatments (limited = 5 g dung for 10 larvae; overabundant = 20 g dung for 10 larvae; cf. Amano, 1983), plus an ivermectin treatment at each temperature and overabundant food treatment combination (a common cattle parasiticide, at concentrations of 6.57 µg/kg dung wet weight; cf. Floate et al., 2016; Römbke et al., 2009). (For the purposes here, ivermectin was merely used as yet another environmental stressor affecting fly life history and reproductive parameters, to supplement our standard food and temperature manipulations.)

In all these studies, we measured egg‐to‐adult development time of all emerged flies of both sexes, their left and right hind tibia lengths as a structural index of body size and to calculate FA (as described below), and we photographed both wings to later score landmarks, wing (centroid) size, and wing FA. In the elaborate study with various treatments at 15 and 23°C, we additionally measured teneral (fresh) body mass of all flies upon emergence. We finally scored the number of spermathecae expressed by all females as a function of their juvenile treatment (after dissection; see Section 2.2).

Our experiments further yielded full‐sib, family‐wise egg‐to‐adult survival rates as the number of adults emerged divided by the number of eggs entered per rearing container. These survival data served to test for a global phenotypic relationship between familial spermathecal expression and juvenile mortality. In the rearings at 24–26.5°C, we embedded an additional pupal mortality assessment to specifically investigate individual effects of 3S versus 4S expression and pupal volume on the probability of individual female emergence (i.e., survival) at extremely high and stressful temperatures (involving opening dead pupae to assess their spermathecal phenotype; see Polak & Tomkins, 2013).

### 2.4 Assessment of wing and leg fluctuating asymmetry

Wing shape was analyzed using 15 landmarks extracted from images photographed by a Leica DM105 light microscope (see Figure 5; cf. Schäfer et al., 2018). Landmarks were digitized using version 2.14 of the software tpsDig2 (Rohlf, 2009), and landmark coordinates were subjected to a full Procrustes analysis using the function gpagen() of the R‐package geomorph, version 3.1.1 (Adams & Otárola‐Castillo, 2013). As a measure of asymmetry for wing shape, we computed the Procrustes distance between the coordinates of the left and the right wing of each individual. That is, we computed the square root of the summed squared distances between corresponding landmarks. This distance represents a measure of asymmetry that is equivalent to the magnitude of a vector that describes the shape differences between an individual's left and right wing (Klingenberg, 2015).

**Figure 2:**
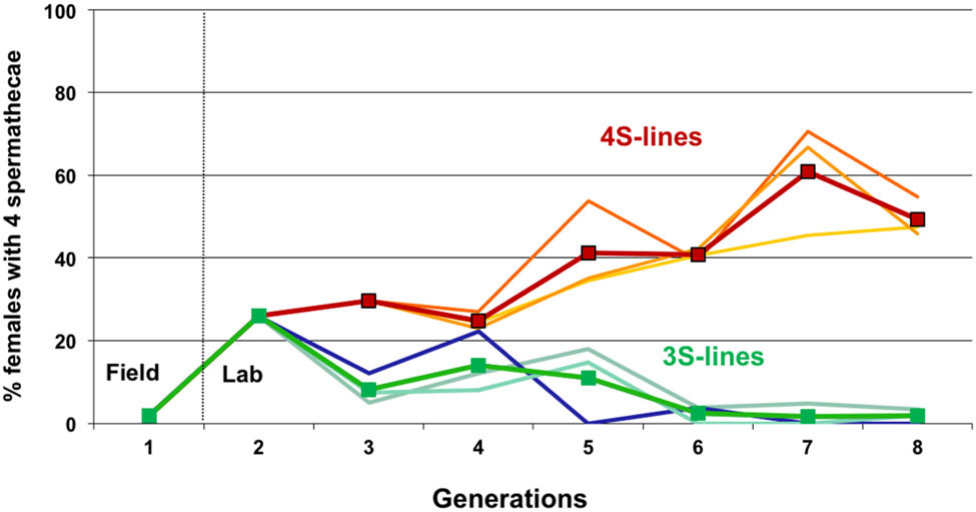
Expression percentage of four spermathecae in the three 4S lines (reddish) and the three 3S lines (greenish) from generation 1 (field situation), via generation 2 (first laboratory‐reared generation), to generation 8 in response to artificial selection in both directions. No unselected control was scored. Bold lines denote the overall average. 4S expression plastically jumps spontaneously from 2% in generation 1 (limited natural field conditions) to ca. 25% in generation 2 (laboratory growth conditions with ample resources), and after eight generations of selection it reaches close to 50% in 4S lines and nearly 0% in 3S lines. Simple line graphs are depicted [Color figure can be viewed at wileyonlinelibrary.com]

Following our earlier FA studies of this species (Blanckenhorn & Hosken, 2003; Hosken et al., 2000), we additionally computed FA of their hind legs in standard ways following Palmer and Strobeck (1986) as: signed FA as (L – R), unsigned FA as (|L – R|) (both in mm), and unsigned, size‐corrected FA as (|L – R|)/mean(L, R).

## 3 RESULTS

### 3.1 Response to artificial selection

The transition from three to four spermathecae occurs in stages: the singlet spermatheca first tends to become heart‐shaped, and successively subdivides to finally form four spermathecae with four fully separated ducts (Figure 1). The percentage of 4S females in the field at generation 1 was only 2% (cf. Berger et al. (2011), who found 3% 4S females in the field), but jumped instantly to ca. 25% in the first laboratory generation 2, a plastic effect presumably mediated by unconstrained growth conditions in the laboratory, to reach roughly 50% after eight generations of artificial selection in the 4S lines (a genetic response; Figure 2). In the 3S lines the percentage of four spermathecae also first jumped up but then diminished toward 1% in generation 8 (Figure 2). The estimated realized heritability of spermathecal expression in the 4S lines across generations 2–8 was *h* ^2^ = 0.637 (*p* < .001; Roff, 1997; cf. Ward et al., 2008).

After eight generations of artificial selection, genetically correlated responses of various other female reproductive traits (mainly internal morphological structures) were evident (depicted in Figure *1* of Thüler et al. [2011]). These traits were separately analyzed using ANCOVAs with selection regime (3S vs. 4S) as fixed factor, lines within selection regime as nested random factor, and body size as single (within‐individual) covariate for most traits (total *N* = 289 females in generation 8, roughly equally distributed among the six replicate lines). Females from 4S lines (mean ± *SE* spermathecal area 0.08329 ± 0.00074 mm^2^) had more but smaller spermathecae than females from 3S lines (0.08776 ± 0.00046 mm^2^), indicating a size‐number trade‐off (selection line effect: *F*_1,4_ = 11.46, *p* = .021), although the total spermathecal storage capacity of 4S females was of course greater. Spermathecal area was positively related to female hind tibia length (overall *r* = .33 based on all females, *p* < .001). At the same time, spermathecal duct length decreased markedly in 4S (0.537 ± 0.0085 mm) relative to 3S lines (0.600 ± 0.0054 mm; selection line effect: *F*_1,4_ = 58.26, *p* < .001; overall correlation with body size *r* = .13, *p* = .046). There were no differences after selection between 3S and 4S lines in female body size (2.68 ± 0.01 mm vs. 2.64 ± 0.01 mm), accessory gland area (0.244 ± 0.0028 mm^2^ vs. 0.238 ± 0.0044 mm^2^), or accessory gland duct length (0.477 ± 0.0050 mm^2^ vs. 0.482 ± 0.0081 mm^2^; all *p* > .2). However, fecundity (first clutch size) of 4S line females (57.0 ± 1.88) tended to be higher than that of 3S females (48.3 ± 1.13; *p* ≈ .1), and clutch size overall slightly decreased with spermathecal size in all lines (partial, size‐corrected *r* = −0.06, *p* = .1).

### 3.2 Spermathecal expression in relation to growth rate

**Figure 3:**
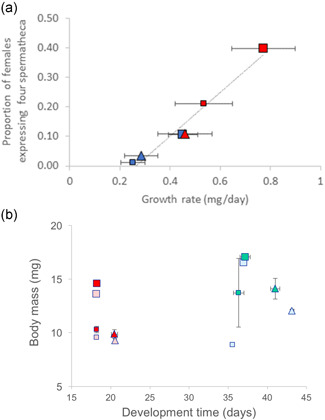
(a) Proportion of females expressing 4 versus the standard 3 spermathecae as a function of growth rate (expressed as fresh body mass/development time ± individual *SD*; *y* = 0.743*x*−0.195, *R* ^2^ = .965, *p* < .001, total *N* = 1487 females; descriptive regression line based on means). (b) Comparison of age and size (mass) at maturity for females with 4 versus 3 spermathecae (dark vs. light symbols, respectively). Symbol color indicates rearing temperature: blue = 15°C, red = 23°C; symbol shape indicates dung treatment: large square = ad libitum, small square = restricted, triangle = ad libitum + ivermectin [Color figure can be viewed at wileyonlinelibrary.com]

Our most elaborate experiment at 15 and 23°C with two dung treatments (limited & abundant) and ivermectin nicely stretched the range of growth rates observed. Across all females (total *N* = 1487), and independent of the precise treatment, the expression of a fourth spermatheca was tightly associated with an increase in growth rate (Figure 3a). Augmented growth of 4S females almost entirely resulted in larger flies, with merely minor variation in development time (Figure 3b). While 4S females were larger than average also within a rearing treatment, body size overall was not a predictor of 4S expression across treatments (Figure 3), as flies became larger when grown in abundant dung but smaller at higher temperatures despite both factors increasing growth rate, as is the case for most ectotherms (Berrigan & Charnov, 1994).

### 3.3 Survival costs of spermathecal expression

**Figure 4:**
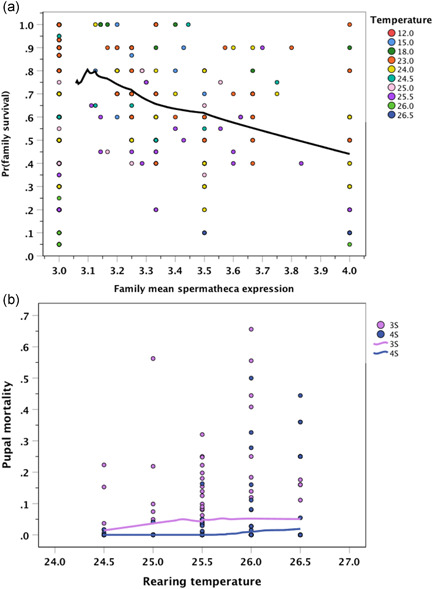
(a) Family‐wise survival rate at any temperature is negatively correlated with the mean frequency of female 4S expression in those families, whether families with only 3S females are excluded (*r* = −.374, *p* < .01; *N* = 192 families) or included (*r* = −.210, *p* < .05; *N* = 507 families). (b) Pupal mortality of 3S versus 4S females at stressfully hot temperatures. Non‐parametric smoothed splines are depicted for description only [Color figure can be viewed at wileyonlinelibrary.com]

Across the entire range of rearing conditions and temperatures (12–27°C), family‐wise egg‐to‐adult survival rates (of both sexes) correlated negatively with the mean frequency of female 4S expression in those families (when ignoring temperature treatment), suggesting life‐history costs of this trait and a trade‐off (simple linear correlation: *r* = −0.210, *p* < .05; *N* = 507 families). This negative relationship remained when including family as random effect (effective *N* = 408 families, because in some data subsets broods were split among temperature environments; see Section 2.3). This result was even clearer when a majority of *N* = 316 families with only 3S females were excluded because they are uninformative in this context (*r* = −0.374, *p* < .01; total *N* = 192 families with variable spermatheca numbers; cf. Figure 4a).

At the same time in a separate experiment, pupal mortality at stressfully high temperatures increased with temperature (24–27°C), as expected (*χ* ^2^ = 13.67, *p* < .001; GLM with binomial errors and sex and temperature as fixed effects; total *N* = 136 pupae). The smaller females generally survived better than the larger males (*χ* ^2^ = 3.84, *p* = .051), an effect that may be (solely?) mediated by their size difference (dimorphism), as pupal size statistically replaced the sex effect in an equivalent model (*χ* ^2^ = 4.03, *p* = .045). In contrast to the overall juvenile egg‐to‐adult mortality results reported above, however, female 4S pupae survived extremely hot temperatures better than 3S pupae except at the highest viable temperature(s) (analogous GLM using only female pupae; spermatheca main effect: *χ* ^2^ = 9.54, *p* = .002; temperature effect: *χ* ^2^ = 23.13, *p* < .001; interaction: *χ* ^2^ = 9.46, *p* = .002; Figure 4b; *N* = 73 female pupae).

### 3.4 Spermathecal expression and fluctuating asymmetry as indicators of developmental instability

**Figure 5:**
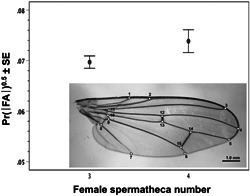
Square‐root‐transformed absolute proportional fluctuating asymmetry (FA) in wing shape of 3S (*N* = 638) versus 4S (*N* = 235) females (*F*_1,871_ = 4.18; *p* = .041) ± *SE*. The wing landmarks used are displayed [Color figure can be viewed at wileyonlinelibrary.com]

Based on a subset of ca. 800 flies of various treatments measured twice, our tibia and wing vein measurements were highly repeatable, as was our FA measurement (*R* > 0.9), which could always be discerned from measurement error (*p* < .001) following Palmer and Strobeck (1986). Absolute (i.e., unsigned) FA of hind tibiae and wings of females (*r* = .038, *p* > .1, *N* = 1562; controlling for body size) and males (*r* = .079, *p* = .003, *N* = 1397) were overall merely weakly positively correlated within individuals, suggesting low general developmental instability in response to diverse environmental stressors (hot temperatures, ivermectin, low food; all flies pooled regardless of treatment). Nevertheless, both tibia and wing FA were positively correlated with growth rate in males (partial *r* = .124 and .111, respectively, both *p* < .001) and, more weakly so, in females (partial *r* = .031, *p* > .1 and *r* = .109, *p* < .001, respectively). Crucially here, wing FA (*F*_1,871_ = 4.18; *p* = .041), but not tibia FA (*F*_1,871_ = .33; *p* = .569), was increased in females expressing four spermathecae (simple GLM comparison of *N* = 638 3S with *N* = 235 4S females raised under various conditions that were ignored in the analysis; Figure 5).

### 3.5 Spermatheca plasticity of a closely related species

**Figure 6:**
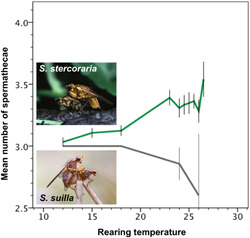
Spermathecal expression increases with temperature in *Scathophaga stercoraria* (green; *N* = 609 females) but decreases with temperature in the closely related *Scathophaga suilla* (gray; *N* = 73 females). Simple line graphs with individual *SE* are depicted [Color figure can be viewed at wileyonlinelibrary.com]

Finally, the increase in the number of spermathecae in *S. stercoraria* associated with faster growth is not a necessary outcome of warmer temperatures, as spermathecal number was observed to decrease with temperature in the closely related *S. suilla* (Figure 6). This striking contrast across species demonstrates that developmental instability does not have a simple directional effect across related taxa, but that these plastic responses, presumably mediated by developmental instability, have evolved in opposing directions within this clade.

## 4 DISCUSSION

The spermathecal polymorphism of yellow dung fly females, which has a demonstrable effect on post‐copulatory sexual and fecundity selection (Berger et al., 2011; Demont et al., 2012, 2021; Schäfer et al., 2013; Simmons et al., 2020; Ward, 2000, 2007), shows a strong heritable component but also exhibits substantial phenotypic plasticity in response to various environmental factors (Figure 2). This equally applies to development and growth rate (Blanckenhorn, 1998, 2009). Our study suggests that any factor that retards or accelerates growth (such as food limitation, warm temperatures, human‐introduced pesticides, etc.) strongly modifies the production during juvenile development of a fourth spermatheca in this species (as opposed to the standard three; Figures 1 and 3). The spontaneous increase in 4S expression from 2% in the field to roughly 25% under overabundant food conditions in the laboratory (Figure 2), the tight relationship of 4S expression with growth rate (Figure 3), and an admittedly weak but positive correlation of 4S expression with both fluctuating asymmetry of wings and legs and growth rate (Figures 3, 5 and 6) suggest that the production of a fourth spermatheca by yellow dung fly females is plastically triggered by any environment conducive to fast growth. We therefore conclude that this natural polymorphism may at least initially be triggered stochastically by likely nonadaptive developmental errors (see also Berger et al., 2011; Schäfer et al., 2013). At the same time, however, we found overall mortality costs, as well as benefits (Figure 4), of 4S pheno‐ and genotypes (also affecting the male siblings), which by contrast suggest a potentially adaptive heritable life‐history trade‐off moderating 4S expression in yellow dung flies (once present). We integrate these contrasting results in our discussion below.

A tight plastic relationship with growth rate (Figure 3) could indicate that the formation of spermathecae is error‐prone during development, potentially induced by pleiotropic relationships with other growth and developmental traits (Berger et al., 2011; Thüler et al., 2011). In this sense, spermatheca expression may be a nonadaptive consequence of developmental instability in contemporary yellow dung fly populations, as has been found and argued occasionally for *D. melanogaster* in the past (Hadorn & Garber, 1944; Mather & Harrison, 1949; Singh et al., 2006; Wexelson, 1928). In *D. melanogaster*, a supernumerary third spermatheca can also occur by loss of function of a particular gene (Gef26; Singh et al., 2006), which is unlikely to be the cause here given our evidence for continuous, environmentally induced evolutionary responses. Our direct evidence for the aberration hypothesis remains limited, however, given merely weak intra‐individual correlations with another common and well‐established indicator of developmental instability, fluctuating asymmetry of paired morphological structures in bilaterally symmetric organisms (FA, here assessed for hind tibiae and wings: Palmer & Strobeck, 1986; Parsons, 1992; Polak, 2003; Van Valen, 1962; Figure 5). In yellow dung flies, FA is not heritable (Blanckenhorn & Hosken, 2003) and a response to hot temperature stress (Hosken et al., 2000), but not responsive to food stress, inbreeding (Hosken et al., 2000), or parasiticide exposure (ivermectin: Floate & Coughlin, 2010). Regardless, 4S females do show greater FA than 3S females, strengthening the hypothesis of a relationship between spermathecal expression and developmental instability (Figure 5). Moreover, the overexpression of female spermathecae at high temperatures is specific, hence perhaps unique to *S. stercoraria*, as in the closely related and ecologically similar *S. suilla* heat exposure reduced rather than increased the number of expressed spermathecae (Figure 6).

As an alternative hypothesis, the observed spermathecal variation might reveal some ancestral form of plasticity that might have been adaptive in the past—for instance, reflecting the optimal solution to a trade‐off between costs of expressing a fourth spermatheca versus the benefits of improved female choice—but which in modern day populations may have become mostly hidden developmental noise exposed only under conditions allowing fast growth. Crucially, however, environmentally mediated plasticity in spermathecal expression, and by extension plasticity in any other morphological, physiological, behavioral, or life‐history character, in most cases changes the stage for natural selection by spontaneously exposing previously unexpressed (genetic) variation (McGuigan & Sgrò, 2009). In *S. stercoraria*, spermathecal and growth rate variation has a (strong) direct heritable component (Figure 2), and may additionally show heritable genotype‐by‐environment (GxE) interactions that can respond to selection in cases of erratic or systematic environmental change (e.g., climate warming; Berger et al., 2011; Blanckenhorn, 1998, 2015; González‐Tokman et al., 2021; Schäfer et al., 2013). Thus, should there be a general advantage in having more (rather than fewer) sperm storing organs (Demont et al., 2012, 2021; Ward, 2000, 2007), then the warmer climes of southern Europe (as shown by Berger et al. [2011]), or other regions now facing climate warming and/or more extensive heat periods in these flies' subarctic ranges (Blanckenhorn et al., 2018; Schäfer et al., 2018), could augment the opportunity for selection on sperm competition and cryptic female choice mechanisms in yellow dung flies (Simmons et al., 2020; Ward, 1998).

By contrast, more ubiquitous pesticides in nature, which generally tend to disturb or at least retard growth (such as the livestock parasiticide ivermectin: Blanckenhorn, Puniamoorthy, Schäfer, et al., 2013; Blanckenhorn, Puniamoorthy, Scheffczyk, et al., 2013; Floate & Coughlin, 2010; Floate et al., 2016; Römbke et al., 2009), would systematically reduce exposure and hence selection imposed on higher spermatheca numbers in these flies. Diminished growth would also be observed in cases of high larval competition due to food (dung) limitation or high fly population densities, even in warmer climes, thus possibly more than offsetting any growth acceleration by warmer temperatures, and instead leading to lower effective expression of the 4S phenotype in nature (Berger et al., 2011; Jann et al., 2000; Schäfer et al., 2013, 2018). In this way, lasting environmental changes of diverse kinds (e.g., when pesticides are introduced: Blanckenhorn, Puniamoorthy, Schäfer, et al., 2013; Blanckenhorn, Puniamoorthy, Scheffczyk, et al., 2013; Puniamoorthy et al., 2014) may contribute to trait diversification, potentially by genetic assimilation (Flatt, 2005; Waddington, 1953; West‐Eberhard, 2003), which ultimately can lead to speciation. The contrasting spermathecal responses to temperature of the two closely related, diversified *S. stercoraria* and *S. suilla* (Figure 6) may be such an example.

Whatever the impact of developmental error in producing the 4S phenotype of *S. stercoraria*, direct evidence that female 4S expression is adaptive remains equivocal even after this study. Fitness differences between the two alternative spermathecal phenotypes are difficult to show by direct experimental comparison in a sexual selection context (Schäfer et al., 2013), as are unambiguous demonstrations of sperm selection by females, regardless of their spermathecal number (Demont et al., 2012, 2021). Somewhat reduced fecundity of 4S females has been reported in previous studies (Ward et al., 2008), whereas our selection experiment here resulted in larger clutches laid by 4S line females, but also in a negative relationship, that is, a potential trade‐off, between a female's spermathecal volume, which was greater in 3S lines, and her clutch size. Moderate egg‐to‐adult survival costs of 4S expression could be demonstrated here (Figure 4a) and before (Schäfer et al., 2013). However, at stressfully high temperatures, at which mortality quickly approaches 100%, 4S female pupae apparently survived better than 3S pupae (Figure 4b), the opposite mortality pattern. These conflicting fitness effects possibly suggest that weak antagonistic pleiotropy across developmental stages and/or environmental contexts may further contribute to the diversification of female sperm storage organs.

Female reproductive structures, including the number of spermathecae, repeatedly have been found to vary considerably within and across even closely related insect taxa (Minder et al., 2005; Pitnick et al., 1999; Presgraves et al., 1999; Puniamoorthy et al., 2010). A preliminary survey shows strong variation between 0 (no sclerotized sperm storing structures whatsoever) and 4 spermathecae across Dipteran families (Table S1), with unsclerotized sperm storing organs (e.g., seminal receptacles) alternatively being present in some species. In light of the above‐described scenario, whether this evolutionary diversification in the end resulted from adaptation by natural selection, or from nonadaptive (random, pleiotropic) side‐effects of standard (temperature‐dependent) metabolic processes, cannot be easily distinguished or reconstructed. Regardless of whether we consider the spermathecal plasticity documented here and elsewhere (Berger et al., 2011; Schäfer et al., 2013) adaptive, its evolution will be mediated by the usual mixture of selective and neutral processes interacting in spatio‐temporally variable environments (i.e., fluctuating‐stabilizing selection; *sensu* Istock, 1981). This is because any short‐term environmental change at an evolutionary, perhaps even ecological time scale may be sufficient to significantly and sustainably change the micro‐evolutionary trajectories of certain traits (e.g., here spermathecal number) in certain altered environments (e.g., by warming climate or human‐mediated pesticides).

## CONFLICT OF INTERESTS

The authors declare that there are no conflict of interests.

## AUTHOR CONTRIBUTIONS

All authors contributed to conceptualizing this study. The MS was written by Wolf U. Blanckenhorn with input from all others. Karin Thüler performed the spermatheca selection experiment under direct supervision of Luc F. Bussière & Wolf U. Blanckenhorn. Richard J. Walters, David Berger, Luc F. Bussière, Wolf U. Blanckenhorn, and Martin A. Schäfer conducted the rearing experiments that generated the survival and fluctuating asymmetry data. Patrick T. Rohner reanalyzed these data using up‐to‐date statistical methods. Ralf Jochmann conducted the comparative survey presented in Table S1.

## Supporting information

Supporting information.

## Data Availability

The various data sets underlying this study can be found in the Supporting Information.
