# Supplementary material for: Growth rate mediates hidden developmental plasticity of female yellow dung fly reproductive morphology in response to environmental stressors
Source: Evol Dev. 2022 Jan 24;24(1-2):3–15. doi: 10.1111/ede.12396 (PMC9285807; doi:10.1111/ede.12396)
Supplement: Supplementary file 1 — Supporting information. [file EDE-24-3-s001.docx]

**Supplementary Table S1:** Preliminary survey of spermathecal variation within the Diptera, based on the standard enzyclopedia *Manual of Palearctic Diptera* plus the primary literature.

**Family No. spermathecae Min. species* Notes**

**Acarthophthalmidae 0?** no sclerotized spermathecae

**Acroceridae ?**

**Agromyzidae 2**

**Anisopodidae 1 or 3 *2*** small and heavily sclerotized

**Anthomyiidae 3 *3***

**Anthomyzidae 2 *3***

**Asilidae >1 *2*** sclerotized, recurved tubes with short duct

**Asteiidae 2**

**Aulacigasteridae 3 *2***

**Axymyiidae ?**

**Bibionidae 3**

**Blephariceridae 3** typically 3, variable in shape

**Bolitophilidae 2**

**Bombyliidae 3**

**Borboropsidae 3 *2***

**Braulidae 1** small and with a pair of small accessory glands

**Calliphoridae all 3 *53***

**Camillidae 2 *1***

**Campichoetidae 2 *3*** sclerotized, rounded or elongate

**Canacidae 2** Variable shape

**Canthyloscelidae 1**

**Carnidae 0?** no sclerotized spermathecae

**Cecidomyiidae 0, 1 or 2 *25*** only some primitive groups have 1 or 2

**Celyphidae 3 *1*** (1+2), globular

**Ceratopogonidae 1, 2 or 3 *11***

**Chamaemyiidae 4 *1***

**Chaoboridae 3** 2 of 3 spermathecal ducts are joined

**Chironomidae 2 or 3 *7*** generally ovoid

**Chiropteromyzidae 2 *2*** semiglobular, flying-saucer-shaped

**Chloropidae 2 *1*** rudimentary

**Chyromyidae 2** spermatecal ducts long and coiled

**Clusiidae 2** orbicular

**Cnemospathidae 3**

**Coelopidae 3?**

**Corethrellidae 1 *1***

**Cremifaniidae 3** globuliform, each with long

**Cryptochetidae 1 or 2**

**Culicidae (1), 2 or 3 *37*** 2 or 3 in most Aedines

**Curtonotinae 2 *3*** short and round

**Cypselosomatidae 2 *1*** probably true for the whole family

**Deuterophlebiidae 0**

**Diastatidae 0 *1***

**Diopsidae 2 or 3 *16*** usually 3, reduced in some genera

**Ditomyiidae 2**

**Dixidae 1 *1***

**Dolichopodidae ?**

**Drosophilidae all 2 *110*** capsule mostly sclerotized, of various shapes

**Dryomyzidae 3 *3***

**Ephydridae 2** usually rudimentary and probably nonfunctional

**Fannidae typ. 3, some 2 *2*** spherical, pear-shaped or elongate

**Fergusoninidae 0**

**Glossinidae 2 *3***

**Heleomyzidae 2 or 3 *5*** more or less globular spermathecae

**Hesperinidae 3**

**Heterocheilidae ?**

**Hilarimorphidae ?**

**Hippoboscidae 2 or 3 *2***

**Hypodermatidae 3**

**Keroplatidae 2 *3***

**Lauxaniidae 3 or 4** globular or ovoid

**Limosininae 3 *1***

**Lonchaeidae ?**

**Lonchopteridae ?**

**Lygistorrhinidae 2**

**Milichiidae 2 *1*** weakly sclerotized and slender

**Muscidae typ. 3, some 2 *8***

**Mycetobiidae 2 *2*** weakly sclerotized

**Mycetophilidae 2 *5***

**Nannodastiidae 0**

**Neriidae 4 *1***

**Nycteribiidae ?**

**Nymphomyiidae 0**

**Odiniidae 2 *2*** usually globular

**Oestridae 3 *1***

**Opetiidae 0?** sclerotized spermathecae are absent

**Opomyzidae 2 *2*** Subspherical or elongate

**Otitidae 3 *1***

**Pallopteridae 2 or 3 *3*** sclerotized, elliptical and with smooth surface

**Pelecorhynchidae 3**

**Phaeomyiidae 3 *1***

**Phoridae 0, 1, 2 or 3** usually unsclerotized and elongated

**Piophilidae 2 *1***

**Pipunculidae 3** well-sclerotized or soft and membraneous

**Platypezidae 3 *2***

**Platystomatidae 3 *2***

**Pseudopomyzidae 2** not known for all spp.

**Psilidae 0?** sclerotized spermathecae absent

**Psychodidae 2 *4***

**Ptychopteridae 3**

**Rachiceridae 1 or 3? *1***

**Rhagionidae ?**

**Rhinophoridae 3 *1***

**Rhinotoridae 2**

**Risidae 0**

**Sarcophagidae 3 or 2 *7*** ovate, pyriform, or elongate

**Scathophagidae typ. 3, some 2, rare 4 *21***

**Scatopsidae 1** well-sclerotized

**Scenopinidae 2** partly sclerotized

**Sciomyzidae 2 *1***

**Sepsidae all 2 *21***

**Simuliidae 1 *4*** mostly well-sclerotized

**Sphaeroceridae 2 or 3 *7*** 3 (most Limosininae) or 2 (Sphaerocerinae, Copromyzinae)

**Stomoxyinae 2**

**Stratiomyidae 0 or 3 *4*** 3 ground plan, completely reduced in some groups

**Streblidae ?**

**Strongylophthalmyiidae 1 *1***

**Syrphidae 3 *1*** small

**Tabanidae 3 *3*** 3S basal feature of Brachycera

**Tachinidae ?**

**Tanypezidae 2 *1***

**Tephritidae at least 1 *9***

**Teratomyzidae 2 *1***

**Tethinidae 2**

**Tipulidae 3**

**Trichoceridae 1 or 3 *7***

**Trixoscelididae 3 *1***

**Xenasteiidae 2 *1***

**Xylomyidae 2 or 3 *6***

**Xylophagidae 3 or 1? *1***

* no number: general information from encyclopedia; otherwise min. number of species group documented
